# Host plant richness and environment in tropical forest transformation systems shape arbuscular mycorrhizal fungal richness

**DOI:** 10.3389/fpls.2022.1004097

**Published:** 2022-10-13

**Authors:** Nur Edy, Henry Novero Barus, Reiner Finkeldey, Andrea Polle

**Affiliations:** ^1^ Forest Botany and Tree Physiology, University of Goettingen, Göttingen, Germany; ^2^ Department of Agrotechnology, Tadulako University, Palu, Indonesia; ^3^ Forest Genetics and Tree Breeding, Göttingen, Germany; ^4^ Center of Biodiversity and Sustainable Land Use, University of Goettingen, Göttingen, Germany

**Keywords:** arbuscular mycorrhiza, diversity, oil palm (*Elaeis guineensis*), rubber tree (*Hevea brasiliensis*), tropical rain forest, plant identification

## Abstract

Transformation of tropical lowland rain forests into rubber tree and oil palm plantations is the cause of massive loss of vegetation diversity. The consequences for associated mycorrhizal fungi are not fully understood. We hypothesized that generalist arbuscular mycorrhizal fungi are resistant to removal of host species richness and that forest conversion to oil palm and rubber leads to loss of arbuscular mycorrhizal fungal (AMF) species with host preferences. Plant identities and AMF species were determined by molecular barcoding of 112 roots collected in three land-use systems (rain forest, rubber tree and oil palm plantation) in two landscapes on Sumatra (Indonesia), a world hotspot of forest transformation. The collected roots were from 43 forest plant species, in addition to rubber trees and oil palms. We detected 28 AMF species of which about 75% were present in forest trees and 25% shared among the land use systems. Only one AMF species present in plantation roots was not detected in the analyzed forest roots. Host specificity of arbuscular mycorrhizal fungi was not detected. Oil palm and rubber tree roots exhibited a strong reduction in AMF richness compared with roots from rainforests and were differentiated by soil resources. On basis of an individual root, oil palm had a lower AMF species richness than forest or rubber tree roots. Our results demonstrate that tropical AMF communities are shaped by two mechanisms: (i) root habitat diversity as the result of plant diversity and (ii) habitat properties as the result of plant traits or environmental conditions and management. Collectively, deterioration of habitat diversity and properties exacerbates impoverishment of AMF assemblages.

## Introduction

Globally, tropical rainforests are replaced at larges scales by agronomic land use systems (Margono et al., 2012). Indonesian lowland rain forests, representing a world hotspot of biodiversity, have undergone particularly drastic transition by agricultural expansion in the past decades (Margono et al., 2012). For example, between 1990 and 2011, forest land cover on Sumatra, Indonesia, decreased from 66% to 37%, while plantation areas with introduced species of oil palm (*Elaeis guineensis*, Jacq.) and rubber trees [*Hevea brasiliensis*, (Willd. ex A. Juss.) Müll. Arg.] increased from 28% to 45% (Clough et al., 2016). This transformation process generates financial gain at the expense of biodiversity and ecosystem multifunctionality (Koh and Wilcove, 2008; Lambin and Meyfroidt, 2010; Grass et al., 2020).

In tropical areas, arbuscular mycorrhizal fungi are keystone species as drivers of ecosystem processes (Powell and Rillig, 2018). They link matter fluxes between the above- and belowground compartment, and thereby, impact plant-environment interactions, plant productivity and ecosystem functions (Van Der Putten, 2009; Klironomos et al., 2011; Wagg et al., 2011; Öpik and Moora, 2012; Heijden et al., 2015). Plants require considerable amounts of phosphorus (P) and nitrogen (N), but most plants have to deal with low N and P concentrations in natural habitats on a regular basis (Ferrol et al., 2019; Kobae, 2019; Balestrini et al., 2020). Arbuscular mycorrhizal hyphal networks contribute to the absorption and translocation of these nutrients from the soil to the host plant, in exchange for host plant-derived carbohydrates (Walder and van der Heijden, 2015). Arbuscular mycorrhizal fungi defend their host from soil-borne pathogens (Singh and Giri, 2017) and environmental stress, in addition to fostering plant growth (Lenoir et al., 2016). Because of their multiple ecosystem functions and services, the impact of tropical land transformation on arbuscular mycorrhizal fungal (AMF) taxonomic structures is receiving increasing attention.

The conversion of lowland rainforests into rubber tree and oil plantations results in massive decrease in vegetation species richness (Rembold et al., 2017). In soils of tropical rainforest transformation systems, overall fungal species richness was hardly affected but a distinct loss of symbiotrophic fungi was observed (Kerfahi et al., 2014; Brinkmann et al., 2019). This reduction was mainly due to the loss of ectomycorrhizal fungi and ascribed to the removal of host tree species (e.g., Dipterocarpaceae, Fagaceae) (Kerfahi et al., 2014). In contrast to ectomycorrhizal fungal species, AMF species show less host specificity (Toju et al., 2014; Heijden et al., 2015) and therefore, may be less sensitive to changes in vegetation composition. However, several investigations discovered that AMF species are selective for host plants (Grime et al., 1987; Miller and Kling, 2000; Bever, 2002; Carrenho et al., 2002; Zhang et al., 2010). Furthermore, AMF communities in different tropical habitats show ecological structures (Rodríguez-Echeverría et al., 2017). Therefore, it is unclear how the replacement of the highly diverse forest vegetation by monocultures of rubber or oil palm trees influences root-residing AMF taxa.

Here, we investigated the association of AMF species with roots of distinct plant species in lowland rain forests, rubber tree and oil palm plantations. We hypothesized that forest conversion leads to loss of specialist and resistance of generalist AMF taxa. We conducted our study in two landscapes on Sumatra (Indonesia) characterized by loam respective clay acrisol soil types (Allen et al., 2015). In each landscape, we collected roots in a national rainforest reservation (Bukit Duabelas National Park and Harapan rainforest), and in oil palm and rubber tree plantations. We used individual roots for the molecular identification of the host plant and associated AMF species. We found that individual forest and rubber tree roots harbored a higher number of AMF species than oil palm roots. Only 25% of the fungal species occurred in all land use systems, while the majority was present only in forest roots. In plantation roots, one AMF species was identified that was not found in the forest roots.

## Materials and methods

### Study sites

We conducted our study in tropical Asia (Jambi province, Sumatra, Indonesia, **Figure S1**). We used two landscapes for sampling, Bukit Duabelas and Harapan rainforest. In Bukit Duabelas, the annual mean temperature is 26.8°C and the precipitation 2860 mm; in Harapan, the annual mean temperature is 26.9°C and the precipitation 2332 mm (Drescher et al., 2016). The landscapes differ in soil conditions. The soils in the Harapan region are loam acrisol soils containing a higher portion of silt and sand and lower stocks of soil C, N and basic cations than the clay acrisol soils in the Bukit region (Allen et al., 2015). In each landscape, three different land use systems were chosen: rain forest, oil palm plantations, and rubber tree plantations. **Table S1** shows where the plots are located geographically. In each plot, three subplots were used for sampling.

### Sampling and root selection

In each subplot, three soil samples (0.20 m depth, 0.04 m diameter) were harvested at a distance of 1 m from a tree. Each sample was kept separately in a plastic bag, placed in a cooling box (Sarstedt, Nümbrecht, Germany) and stored 4°C (University of Jambi, Jambi, Indonesia). From each plastic bag, one single root was taken randomly, cleaned with tap water, and freeze-dried (Benchtop K, VirTis, SP Industries, Gardiner, NY, USA). The dry roots were send to the University of Göttingen (Göttingen, Germany). Sampling, export and import permissions according to the Biodiversity Convention have been reported in the Supplemental materials (**Figure S1**).

### DNA extraction and PCR for AMF species

We used 112 roots (36 from rain forest, 10 from oil palm and 10 from rubber trees per landscape) for DNA extraction with the innuPREP plant DNA kit (Analytik Jena, Jena, Germany) according to the manufacturer’s protocol. The DNA concentration used for polymerase chain reaction (PCR) was 50 ng in 2 µl. We conduced a nested PCR using general fungal primers NS1 and NS4 (White et al., 1990), followed by a second PCR with AMF-specific primers AML1, and AML2 modified after (Lee et al., 2008). The technical details of the PCRs have been reported in the supplemental materials (**Supplemental Method 1**) and the primer sequences are shown in **Table S2**. Eight clones of each root sample were sequenced by a company (SEQLAB Sequence Laboratories Göttingen GmbH, Göttingen, Germany).

We used the same DNA extracts, which had been prepared from individual roots (see above) for fungal analysis, for plant species identification. We applied the barcoding markers *rbcL* and *matK* (Kress et al., 2009; Yu et al., 2011) (**Table S2**), as recommend by the Consortium for the Bar Code of Life (CBOL Plant Working Group et al., 2009; de Vere et al., 2015). The technical details are shown in the **Supplemental Method 1**. Sequencing was conducted in-house (Applied Biosystems 3130xl Genetic Analyzer, Life Technologies GmbH, Darmstadt, Germany). The PCR products of *matK* were rarely successful and therefore, analysis of plant sequences was based mainly based on the marker gene *rcbL*.

### Sequence analysis

DNA sequences were edited using BioEdit (Hall, 1999) and aligned with MEGA 6 (Tamura et al., 2013). AMF species were searched by BLAST in the MaarjAM (Öpik et al., 2010) and the NCBI data bases (Sequeira, 2013). Plant sequences were BLAST-ed against the NCBI database and verified with BOLD Systems (Ratnasingham and Hebert, 2007). We assigned species names when the sequence identity was >97%, a widely used threshold for fungal barcoding (Toju et al., 2014). Details of the molecular identification are shown for plants in **Tables S3, S4** and for arbuscular mycorrhizal fungi in **Tables S5, S6**. AMF species and their plant host sequences have been deposited in NCBI Genbank (accession numbers: KR822761 to KR822789 and MH412812 to MH412923, respectively).

### Statistical analyses

A data matrix was set up containing host plant identities (**Tables S3**, **S4**), AMF species per distinct host root (**Tables S5**, **S6**) and soil nutrients (C, N, C/N, available P, K, Mg, Ca). The soil nutrient data were measured in our plots by Sahner et al. (2015) and downloaded from Dryad (https://doi.org/10.5061/dryad.qf362). We used the fungal data to generate rarefaction curves, to calculate diversity indices (measured species richness, Chao1, Simpson index, Shannon diversity H´, and evenness = e^H/S^), and for analyses of similarity (PERMANOVA) in PAST 3.02 (Hammer et al., 2001). Non-metric multidimensional scaling based on Bray Curtis similarities was used to show AMF community structures. Soil parameters were fitted in PAST and significant parameters (p< 0.05) were indicated by vectors. Differences between count data for species were analyzed with Poisson distribution and a *post-hoc* test (Wilcoxon) and comparisons of regression curves were conducted with Statgraphics 18 Centurion (Statgraphics Technologies, Inc., The Plains, Virginia, USA). Venn diagrams were constructed using BioVenn to demonstrate overlap between AMF species across different types of land uses (Hulsen et al., 2008). The d’ index of specialization was used to calculate the specificity of AMF species to the plant host (Blüthgen et al., 2007). Network analysis (R Core Team, 2020) was used to visualize the distribution of AM species among different host plant species using the “bipartite” v2.05 package with the “plotweb” function in R (Dormann et al., 2009). ClustVis (http://biit.cs.ut.ee/clustvis) was used to create heat maps for fungal patterns in plant roots (Metsalu and Vilo, 2015).

## Results

### Host plant and AMF species richness

**Figure 1:**
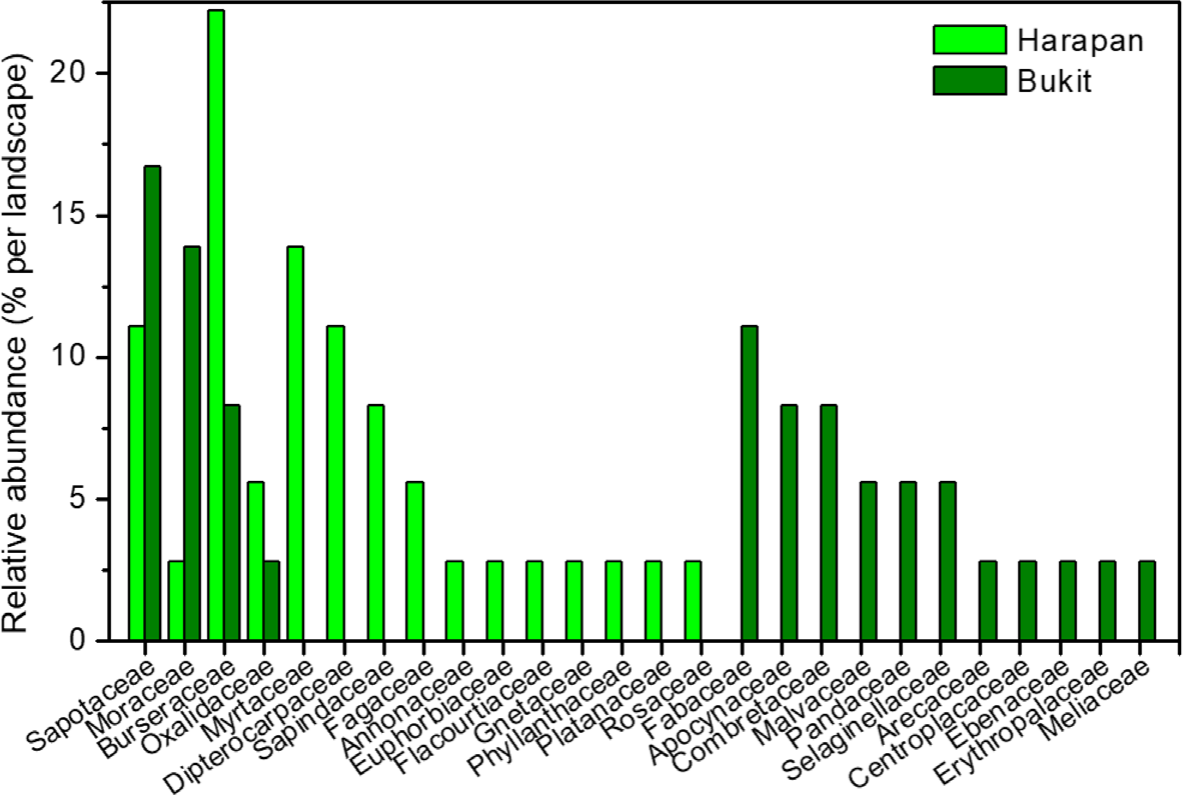
Relative abundance of plant families in Harapan and Bukit. Numbers indicate percentage per plant family and landscape.

We used a total of 112 roots from two landscapes (H = Harapan, B = Bukit Duabelas) and three land use systems (F = forest, R = rubber tree plantation, O = oil palm plantation) for plant and AMF species identification (**Tables S3**–**S6**). Roots from BF (n = 36) belonged to 18 plant species in 15 families and roots from HF (n = 36) to 29 plant species in 15 families (**Figure 1**). Only four plant species, each from another plant family, were common to the samples from the two landscapes analyzed here (*Canarium ovatum*, [Engl.]*, Dapania racemosa*, [Korth.]*, Micropholis garciniifolia*, [Pierre]*, Micropholis longipedicellata* [Aubrév]). In each landscape, the shared plant species comprised together about 42% of the analyzed roots. Rubber and oil palm trees were represented by all roots collected from plantations (10 per landscape and plantation) (**Tables S3, S4**).

**Figure 2:**
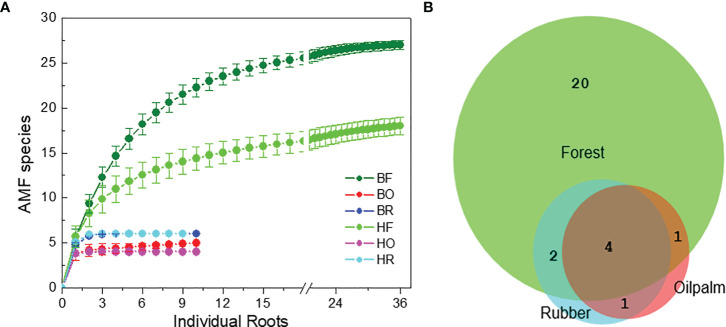
Rarefaction curves for arbuscular mycorrhizal fungi **(A)** in rain forest (HF, BF), rubber tree (HR, BR), and oil palm (BO, HO) plantations in two landscapes (B = Bukit, H = Harapan) and overlap of the fungal species among the land use systems **(B)**.

We examined an average of eight clones per root for AMF identification (896 clones in total). We discovered 28 AMF taxa (**Tables S5, S6**). The detected AMF species belonged to following fungal families: Glomeraceae (62%), Acaulosporaceae (15%), Racocetraceae (7%), Diversisporaceae (5%), Archaeosporaceae (3%), Ambisporaceae (3%), Claroideoglomeraceae (3%), and Dentiscutataceae (1%) families (**Tables S5, S6**). *Glomus* was the most common genus. At the specified sample and sequencing depth, rarefaction curves revealed saturation for AMF richness (**Figure 2A**).

Among the AMF species, 20 taxa were unique to forest roots, 7 shared among forest and plantation roots and one species (OTU25 = *Glomus* sp. Voyria VTX00126, **Supplement Tables S5**, **S6**) was only present in roots of oil palms and rubber trees (**Figure 2B**). Four AMF species were shared among all land use systems (OTU1 = *Acaulospora lacunosa* [J.B. Morton] VTX00024, OTU2 = *Acaulospora lacunosa* 2, OTU20 = *Glomus* sp. VTX00194, OTU23 = *Glomus* sp. VTX00363) (**Figure 2B** and **Supplement Tables S5, S6**).

### AMF diversity and community structures

**Figure 3:**
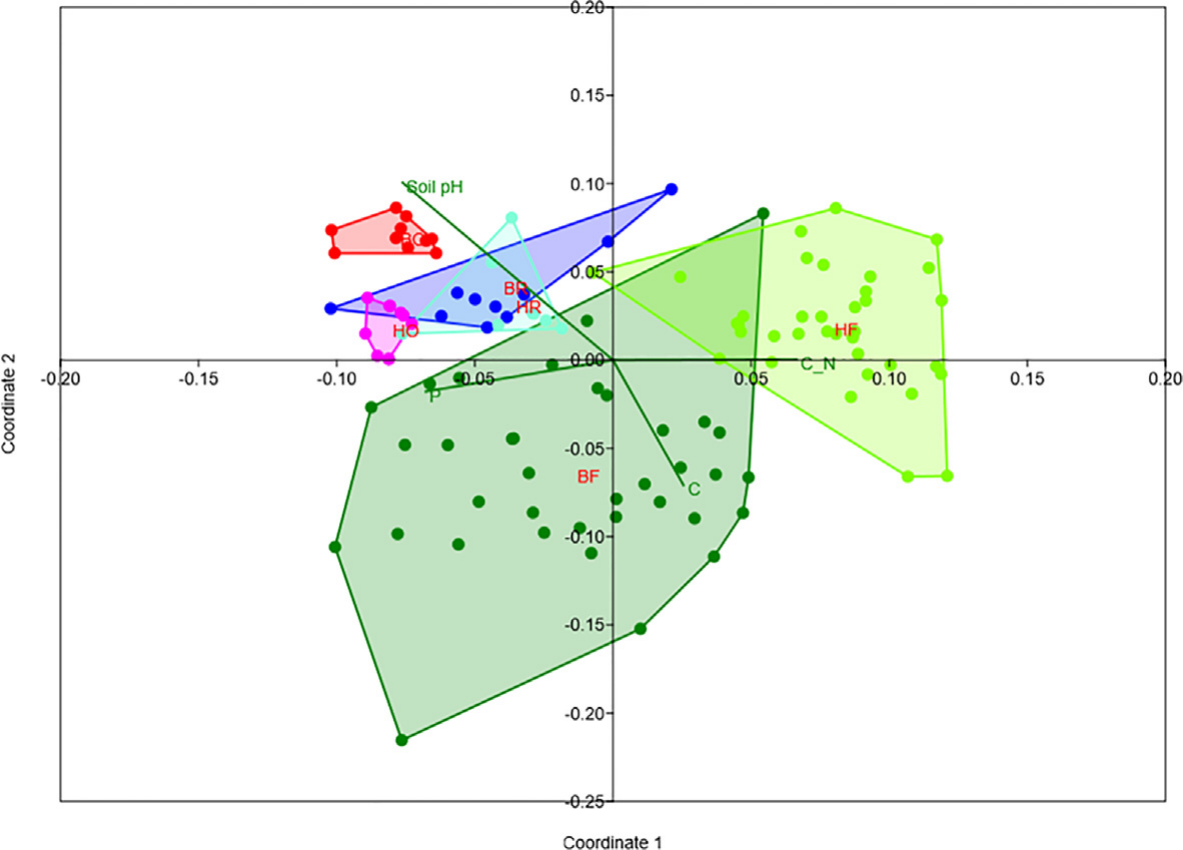
Non-metric multidimensional scaling of arbuscular mycorrhizal fungi in rain forest (HF, BF), rubber tree (HR, BR), and oil palm (BO, HO) plantations in two landscapes (B = Bukit, H = Harapan) and fit of significant environmental variables (soil pH, available soil phosphorus = P, soil carbon = C, C_N = C/N ratio). Stress = 0.18.

Rubber trees and oil palms in Bukit Dubelas and Harapan exhibited similar AMF species richness, Shannon diversity and high evenness (**Table 1**), while rainforests (using the same numbers of analyzed roots as in the plantations) showed higher AMF species richness and Shannon diversity but lower evenness (**Table 1**). Ordination revealed separation of the AMF community structures in the three land use systems (**Figure 3**). PERMANOVA showed that AMF structures in the two landscapes were also distinct (**Table 2**). Differences between the AMF community structures in forests and plantations were mainly driven by soil carbon and pH differences, whereas the AMF structures in the two landscapes were driven by higher soil C/N (Harapan forest) and higher available phosphorus in soil (Bukit Duabelas forest) (**Figure 3**).

**Table 1:** Diversity indices for arbuscular mycorrhizal fungi in rainforest, rubber, and oil palm roots collected in two landscapes (Harapan, Bukit Dubelas).

	BF	HF	BR	HR	BO	HO
Species richness	23	14	6	6	5	4
Chao1	32	16	6	6	5	4
Simpson	0.93	0.90	0.83	0.83	0.76	0.75
Shannon H´	2.88	2.41	1.78	1.79	1.47	1.38
Evenness e^H/S^	0.77	0.80	0.99	0.99	0.87	1.00

BF, forest in Bukit Duabelas; BO, oil palm plantation in Bukit; BR, rubber tree plantation in Bukit Duabelas; HF, forest in Harapan; HO, oil palm plantation in Harapan; and HR, rubber tree plantation in Harapan. Richness show cumulated data based on 10 roots per landscape and land use system. For BF and HF cumulated richness data of 10 roots were averaged (n = 3).

**Table 2:** Permutational multivariate analysis of variance of arbuscular mycorrhiza in plant hosts along land use systems in the landscapes Bukit and Harapan.

**Source**	**Sum of sqrs**	**Df**	**Mean square**	**F**	**p**
Landscape	0.321	1	0.321	1.813	0.019
Land Use	4.191	2	2.096	11.847	<0.001
Interaction	2.367	2	1.183	6.689	0.423
Residual	5.130	29	0.177		
Total	7.276	34			

The land use systems are plots in forest, oil palm and rubber tree plantations. Analysis was conducted with 9999 permutations using Bray Curtis as the similarity measure.

### Arbuscular mycorrhizal fungi and host associations

**Figure 4:**
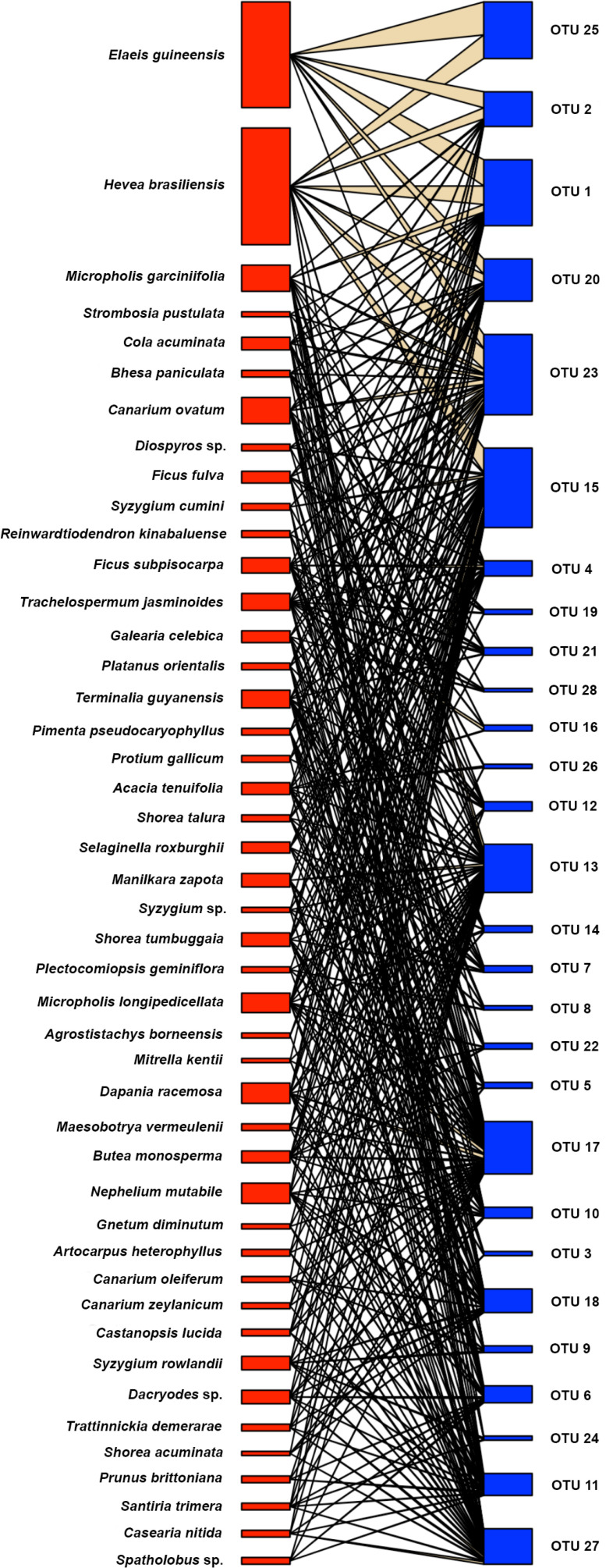
Association between plant species (red) and arbuscular mycorrhizal fungal species (blue) determined by bipartite modelling. Thickness of boxes indicates abundance of fungal and plant species.

A bipartite network plot clustering the plant species according to similarity was used to illustrate the quantity and dispersion of AMF species in their host plants (**Figures 4** and **S2**). The bipartite network indicated a higher number of associations between plant and AMF taxa for forest species than for plantation species (**Figure 4**). Host association specificity has d’ values ranging from 0 (generalization) to 1 (specialization) (Blüthgen et al., 2007). Here, we found the d’ indices ranged from 0.17 to 0.50, thus, not supporting specialization of AMF species. However, the four fungi, which were shared among all land use systems and contributed 34% to total fungal abundances, had a lower mean d´ (0.20 ± 0.03) compared to that of the other AMF species (d´= 0.31 ± 0.08, *p* = 0.012). Clustering patterns of the AMF species in individual roots underpinned similarities between rubber tree and oil palm AMF taxa and differences between the forest taxa in Harapan and Bukit Dubelas (**Figure S2**).

**Figure 5:**
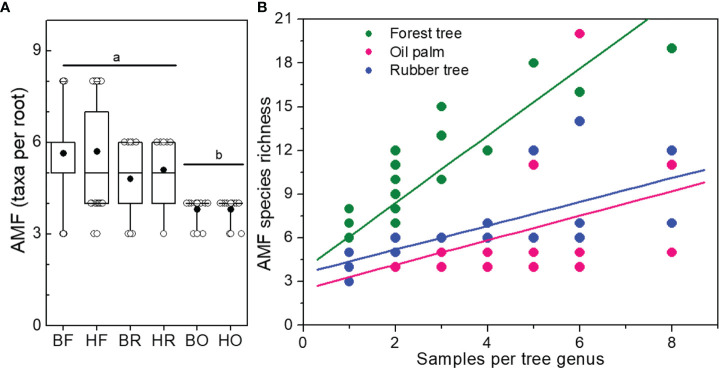
Number of different arbuscular mycorrhizal fungal species (AMF) per root **(A)** and per sampling intensity of tree genera **(B)**. Boxes and whiskers indicate range of data (25 to 75% and 95%, respectively), median = horizontal line, mean = black circle. Rain forest (HF, BF), rubber tree (HR, BR), and oil palm (BO, HO) plantations in two landscapes (B = Bukit, H = Harapan). Different letters above the box plots indicate significant differences at *p* < 0.05. Linear models were applied to draw regression curves.

We found higher AMF species richness in Bukit than in Harapan forest roots (**Figure 2B** and **Table 1**) although plant species richness of the analyzed roots was lower in Bukit than in Harapan forest (**Tables S3, S4**). The richness of AMF taxa per root was higher in forest and rubber tree roots than in oil palm roots (Wilcoxon test, Forest: *p* = 3.3 x10^-8^, Rubber: *p* = 0.009, **Figure 5**). The difference in AMF richness per single root between forest and rubber trees was marginal (Wilcoxon test, *p* = 0.075). While land use systems influenced the number of fungal taxa per root (Poisson, Chi^2^ = 11.49, *p* = 0.003), landscape had no effect (Chi^2^ = 0.04, *p* = 0.836) (**Figure 5A**).

We tested the relationship between tree genus and AMF species richness (**Figure 5B**). Since the genera of forest trees were represented by one to eight samples, we analyzed AMF richness in the plantation roots also for one to eight samples, using random samples for roots of rubber trees as well as for oil palms. AMF richness increased with increasing numbers of samples per genus (forest genera: ${\text{R}}_{\text{adj}}^{2}=0.776,p<0.001$ ; rubber trees: ${\text{R}}_{\text{adj}}^{2}=0.389,p=0.002$ ; oil palms: ${\text{R}}_{\text{adj}}^{2}=0.169,p=0.041$ ). The slopes and intercepts of the curves for AMF species richness in oil palms and rubber trees did not differ significantly (*p*_intercept_ = 0.291, *p*_slope_ = 0.959). Roots of forest tree genera contained higher AMF species richness that those of the plantation trees (differences for intercept and slopes: *p*_intercept_< 0.001, *p*_slope_< 0.001).

## Discussion

We examined the effect of tropical land transformation on arbuscular mycorrhizal fungi residing in roots. We found that conversion of rainforest into rubber tree or oil palm plantations altered AMF community composition and strongly decreased overall AMF species richness in roots of plantation trees. This result was not only caused by higher plant species richness but also by a higher richness of AMF species per tree genus in the forest. This result adds to the growing awareness that conversion of unmanaged ecosystems into intense agricultural mono-cropping systems in different regions worldwide strongly affects AMF structures (Xiang et al., 2014; Liu et al., 2015; Xu et al., 2017). In soils of south-east Asian forest conversion systems (Borneo, Sumatra, Malaysia) massive shifts in whole fungal community composition were found but the relative abundance of Glomeromycota appeared to be little affected (Kerfahi et al., 2014; McGuire et al., 2015; Brinkmann et al., 2019). For example, McGuire et al. (2015) found an overall decrease in fungal species richness (-30%) in plantation soil but a higher abundance of Glomerales in soils of oil palms than in primary or regeneration forests, while other studies using similar fungal amplicon-based sequencing techniques found no clear effects on AMF abundances (Kerfahi et al., 2014; Brinkmann et al., 2019; Ballauff et al., 2020).

The number of AMF taxa detected in roots depends on the sequencing strategy and bioinformatic filtering as outlined elsewhere (Davison et al., 2015; Vasar et al., 2017; Kajihara et al., 2022). Sequencing depths by Illumina or pyrosequencing is higher than by cloning sequencing used here. However, fungal richness of our study (28 potential AMF species) was similar to that in high throughput studies, reporting about 15 to 30 AMF taxa in roots (Vasar et al., 2017) or up to approximately 30 taxa in tropical forest roots (Davison et al., 2015). Although we reached AMF species saturation in the collected root samples, our study covered only a small fraction of forest plant richness. Our plots contained on average approximately 210 (Harapan) and 270 (Bukit) different plant species (Rembold et al., 2017). Clearly, further studies are required to cover AMF richness in the hyperdiverse tropical rain forests. Nevertheless, we identified typical taxonomic structures of the AMF communities with the highest abundance of species in the genus *Glomus* and lower abundances of other fungal genera or families (Lovelock and Ewel, 2005; Davison et al., 2015; Vasar et al., 2017; Maciel Rabelo Pereira et al., 2020). Our plantation roots exhibited one unique AMF species (OTU25, *Glomus* sp.). One possibility is that this particular species is associated with Arecaceae and Euphorbiaceae and, thus, might be present in rainforest trees from the same families as oil palms and rubber trees. Another possibility is that OTU25 occurs preferentially in managed land use systems, exposed to fertilizers and herbicides. Thus, despite some limitations, our study clearly demonstrates significant differences among the AMF communities in forests and plantations.

The degree of specialization of arbuscular mycorrhizal fungi is a matter of debate. Several studies found selectivity of AMF species for host plants (Grime et al., 1987; Miller and Kling, 2000; Bever, 2002; Carrenho et al., 2002; Zhang et al., 2010), while others reported a lack of host specificity (Clapp et al., 1995; Santos et al., 2006; Torrecillas et al., 2012). For example, a deep sequencing effort of AMF species in roots from five native and one introduced plant species in a remnant and a restored forest on Hawaii resulted in the identification of 1766 amplicon sequencing variants (ASV) (Kajihara et al., 2022). While ASVs are likely overestimating AMF species, their d´ values for specialization ranged from 0.2 to 0.5 in the roots of the analyzed plant species (Kajihara et al., 2022), i.e., were in a range similar to the values found here. In restored forests, d´ was slightly – but significantly – lower than in forest remnants (Kajihara et al., 2022), thus, also resembling the effects found here with lower d´ (0.2) for ubiquitous AMF taxa and higher d´ (0.3) for AMF taxa associated only to forest plants. Since these d´ values are closer to zero (no specialization) than to 1 (specialization) our data agree with the results of meta-analyses that AMF species may have a preference for certain plant communities rather than plant species (Hoeksema et al., 2010; Yang et al., 2012).

In addition to plant diversity, there is growing evidence that AMF specificity and community structure are linked to environmental resources (Carrenho et al., 2002; Hart et al., 2003; Martínez-García and Pugnaire, 2011; Yang et al., 2015) such as carbon, nitrogen, and phosphorus (Bohrer et al., 2001; Carrenho et al., 2007; Hoeksema et al., 2010; Vasco-Palacios et al., 2020). In tropical land-use systems, turnover of soil resources influenced the turn-over of mycorrhizas in roots (Ballauff et al., 2021). In agreement with those results, we found that soil pH and soil carbon separated the AMF community structures in plantation and rain forest roots and that soil fertility (phosphorus and C/N ratio) explained the differences between the two rain forest reservations. These results concur with experimental fertilization that led to decreased arbuscular mycorrhizal fungi in tropical forests (Sheldrake et al., 2017) and increased AMF abundances in oil palm plantations upon reduced fertilization (Ryadin et al., 2022). Thus, fertilization and pesticide treatments were likely factors contributing to AMF species reductions in the plantation trees, resulting in lower AMF richness per genus in oil palm and rubber trees than in forest tree genera. An intriguing question is whether decreased AMF species richness might be related to decreased root colonization. A previous study on the same plots showed that the colonization rates of roots of rubber trees were similar to those of forest trees, whereas oil palm roots showed variable patterns with lower or similar colonization rates (Sahner et al., 2015).

An important novel result was that rain forest and rubber trees harbored higher AMF richness per root than oil palm roots, although overall AMF richness of rubber tree roots was almost as low as in oil plantations. Since soils in rubber tree and oil palm plantations were more comparable than in forests, greater AMF richness in rubber tree and forest roots than in oil palm roots shows that plant features played a role in mediating AMF richness as well. As a result, our findings show that root characteristics influence the habitat of AMF species.

In conclusion, our study shows that forest roots harbor a wide range of AMF taxa. The majority of these forest species is lost after conversion into mono-cropping plantations of rubber trees or oil palms. The loss of taxa present in forest tree roots and the resistance of the most abundant taxa across the land use systems underpins that the land use changes foster generalist AMF communities. We showed that vegetation richness is an important driver for AMF species richness although the association between a distinct AMF species and plant species was moderate. Our study supports that AMF composition reflects ecological signatures as the result of diverse plant traits, soil and root resources. The loss of biological heritage, here represented by AMF species richness, is a matter of concern because vanished species cannot be recovered and the consequences of their loss for long-term ecosystem stability remain unknown.

## Data availability statement

The datasets presented in this study can be found in online repositories. The names of the repository/repositories and accession number(s) can be found in the article/**Supplementary Material**.

## Author contributions

Conceptualization: AP, NE, RF. Field work NE and HB. Laboratory work: NE. Data analysis: NE, AP. Data curation NE, AP. Writing original draft: NE. Writing-review and editing: AP. Funding acquisition: AP. All authors contributed to the article and approved the submitted version.

## Funding

This study was funded by the Deutsche Forschungsgemeinschaft (DFG, German Research Foundation) – project number 192626868 – SFB 990 and the Ministry of Education, Culture, Research, and Technology Indonesia) in the framework of the collaborative German – Indonesian research project CRC990: Ecological and Socioeconomic Functions of Tropical Lowland Rainforest Transformation Systems (Sumatra, Indonesia) project B07. Erasmus Mundus, EURASIA2 provided a scholarship to NE. The CRC990 management board (Prof. Scheu) awarded an internal ABS research grant to NE. The publication was supported by the open access publication fund of the Georg-August Universität Göttingen.

## Acknowledgments

We appreciate the excellent technical assistance of M. Fastenrath. We are grateful to village leaders, local plot owners, PT Humusindo, PT REKI, PT Perkebunan Nusantara VI, and Bukit Duabelas National Park for allowing us access to do research in the study sites. We appreciate Bambang Irawan, Efi Toding, and Sri Wilarso Budi assistance with administrative problems. AP gratefully acknowledges the funding provided by the Deutsche Forschungsgemeinschaft to CRC990 (Ecological and Socioeconomic Functions of Tropical Lowland Rainforest Transformation Systems [Sumatra, Indonesia]) subproject BO7. NE thanks Erasmus Mundus Eurasia 2 for a PhD scholarship and the Tadulako University for allowing him to take a study leave.

## Conflict of interest

The authors declare that the research was conducted in the absence of any commercial or financial relationships that could be construed as a potential conflict of interest.

## Publisher’s note

All claims expressed in this article are solely those of the authors and do not necessarily represent those of their affiliated organizations, or those of the publisher, the editors and the reviewers. Any product that may be evaluated in this article, or claim that may be made by its manufacturer, is not guaranteed or endorsed by the publisher.
