# Supplementary material for: Host plant richness and environment in tropical forest transformation systems shape arbuscular mycorrhizal fungal richness
Source: Front Plant Sci. 2022 Oct 13;13:1004097. doi: 10.3389/fpls.2022.1004097 (PMC9606760; doi:10.3389/fpls.2022.1004097)
Supplement: Supplementary file 1 [file DataSheet_1.pdf]

Supplementary Material

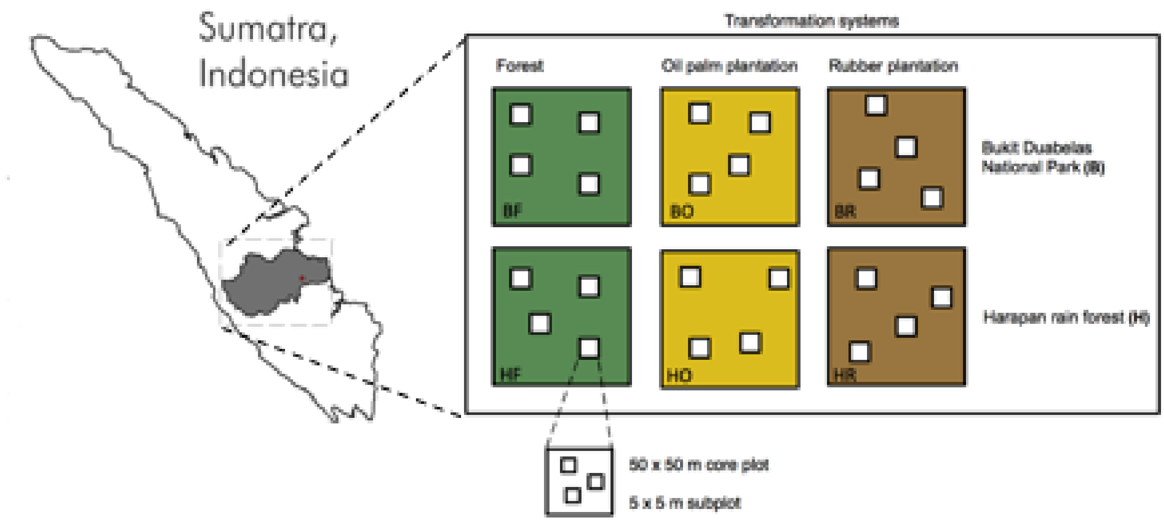

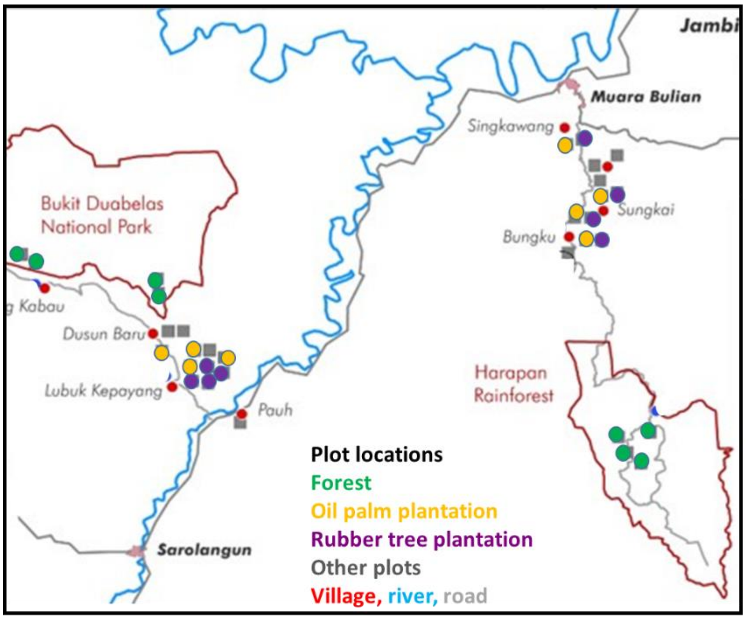

Supplementary Figure 1. Location and design of the study plots in two landscapes (Bukit – B, Harapan – H) in lowland rainforests, oil palm and rubber plantations in the region Jambi on Sumatera (Indonesia). The geographic coordinates are shown in Table S1. We obtained the following sampling and export permissions: The entry permit to conservation area was issued by the National Park of Bukit Duabelas Office (Balai Taman Nasional Bukit Duabelas: Surat Izin Memasuki Kawasan Konservasi [SIMAKSI], number: SI.71/BTNBD-1/2013). The entry to Harapan rain forest was approved by the PT. Restorasi Ekosistem Indonesia (REKI) via email communication between Collaborative Research Center (CRC) office in Universitas Jambi and PT. REKI. The Research Center for Biology of the Indonesian Institute of Science (LIPI: Lembaga Ilmu Pengetahuan Indonesia, Jakarta, Indonesia) issued a sample collection permit (Rekomendasi Ijin Pengamblian dan Angkut [SAT-DN] Sampel Tanah dan Akar, number: 2696/IPH.1/KS:02/XI/2012) for domestic transportation. Recommendation for export permit (number: S.16/KKH-2/2013, Rekomendasi Ijin Membawa/ Mengirim Sampel Tanah dan Akar ke Jerman [SAT-LN], number: 2538/IPH.I/KS.01/XII/2013) was also issued by LIPI. Export permit (reference number: 48/KKH-5/TRP/2014) for all samples from the plot was issued by the Directorate General of Forest Protection and Nature Conservation PHKA (Perlindungan Hutan dan Konservasi Alam, Jakarta, Indonesia) under the Ministry of Forestry of the Republic of Indonesia. The agency for Agricultural Quarantine under the Ministry of Forestry of the Republic of Indonesia certified the samples with “phytosanitary certificate” (reference number: 2013.2.10.03. K10.E000014). The Chamber of Agriculture of Lower Saxony (Plant Protection Office, Hannover, Germany) issued the import permits (Letter of Authority, numbers: DE-NI-12- 69 -2008-61-EC, DE-NI-14- 08 -2008-61-EC).

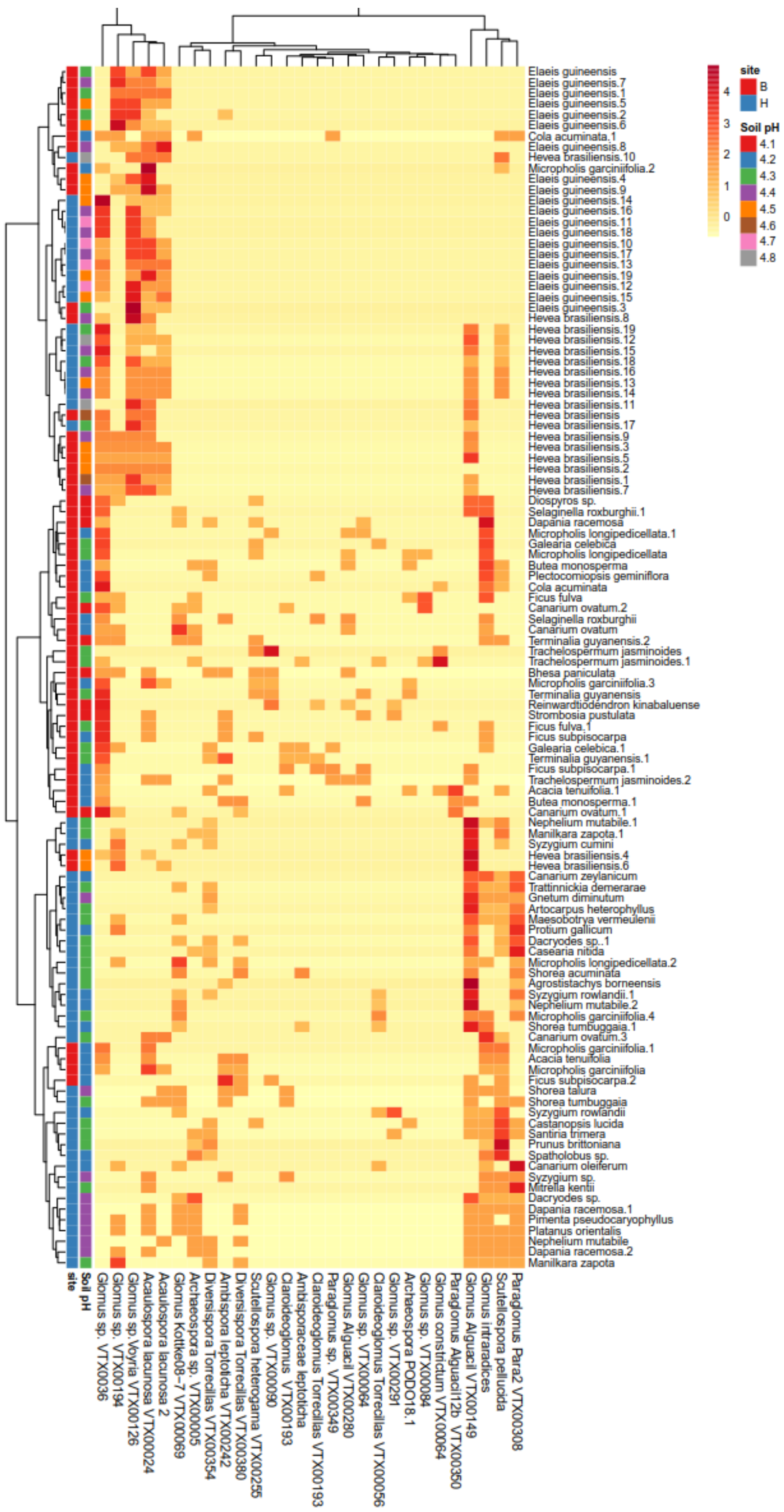

Supplementary Figure 2. Heatmap showing hierarchical clustering of arbuscular mycorrhizal fungi associated plant species. Left columns: Site = blue: Harapan, red: Bukit. Soil pH was categorized in steps of 0.1 units.

Supplementary Table 1. Plot location in Bukit Duabelas National Park and Harapan land use systems

Transformation systems	Plot code	Latitude	Longitude	Altitude (m)
Landscape: Bukit Duabelas				
Rain forest	BF1	S 01°59'42.5"	E 102°45'08.1"	83
Rain forest	BF2	S 01° 58'55.1"	E 102°45'02.7"	77
Rain forest	BF3	S 01°56'33.9"'	E 102°34'52.7"'	87
Rain forest	BF4	S 01°56'31.0"'	E 102°34'50.3"	87
Oil palm plantation	BO1	S 02°04'26.1"	E 102°48'55.1"	75
Oil palm plantation	BO2	S 02°04'32.0"	E 102°47'30.7"	84
Oil palm plantation	BO3	S 02°04'15.2"	E 102°47'30.6"	71
Oil palm plantation	BO4	S 02°03'01.5"	E 102°45'12.1"	34
Rubber tree plantation	BR1	S 02°05'30.7"	E 102°48'30.7"	71
Rubber tree plantation	BR2	S 02°05'06.8"	E 102°47'20.7"	95
Rubber tree plantation	BR3	S 02°05'43.0"	E 102°46'59.6"	90
Rubber tree plantation	BR4	S 02°04'36.1"	E 102°46'22.3"	51
Landscape: Harapan				
Rain forest	HF1	S 02°09'09.9"	E 103°21'43.2"	76
Rain forest	HF2	S 02°09'29.4"	E 103°20'01.5"	75
Rain forest	HF3	S 02°10'30.1"	E 103°19'57.8"	58
Rain forest	HF4	S 02°11'15.2"	E 103°20'33.4"	77
Oil palm plantation	HO1	S 01°54'35.6"	E 103°15'58.3"	81
Oil palm plantation	HO2	S 01°53'00.7"	E 103°16'03.6"	55
Oil palm plantation	HO3	S 01°51'28.4"	E 103°18'27.4"	64
Oil palm plantation	HO4	S 01°47'12.7"	E 103°16'14.0"	48
Rubber tree plantation	HR1	S 01°54'39.5"	E 103°16'00.1"	77
Rubber tree plantation	HR2	S 01°52'44.5"	E 103°16'28.4"	59
Rubber tree plantation	HR3	S 01°51'34.8"	E 103°18'02.1"	90
Rubber tree plantation	HR4	S 01°48'18.2"	E103°15'52.0"	71

Supplementary Table 2. Details of primers used in this study.

Primer	Sequence (5'–3')	Target group	Source
NS1	GTAGTCATATGCTTGTCTC	Fungal (18S rRNA gene)	White et al. (1990)
NS4	CTTCCGTCAATTCCTTTAAG		
AML1	ATCAACTTTCGATGGTAGGATAGA	All AMF groups	Lee et al. (2008)
AML2	GAACCCAAACACTTTGGTTTCC		
M13-20	CGACGTTGTAAAACGACGGCCAGT	General primer for sequencing	pGEM-T Easy vector primers
M13 RP	TTTCACACAGGAAACAGCTATGAC		
rbcLaf	ATGTCACCACAAACAGAGACTAAAGC	Land plants (ribulose-bisphosphate carboxylase gene)	Kress et al. (2009)
rbcLar2	GAAACGGTCTCTCCAACGCAT		
MatKnewF	GTTCAAACTCTTCGCTACTGG	Land plants (chloroplast maturase K gene)	Kress et al. (2009), Yu et al. (2011)
MatKnewR	GAGGATCCACTGTAATAATGAG		
3FKim(MatK)	CGTACAGTACTTTTGTGTTTACGAG		
1RKim(MatK)	ACCCAGTCCATCTGGAAATCTTGGTTC		

AMF: arbuscular mycorrhizal fungi

Supplementary Table 3. Molecular identification of arbuscular mycorrhizal plant host species in Bukit Duabelas National Park transformation systems. Similarity refers to species.

Plot ID	Family	Genus	Species	E-Value	Similarity
**Forest**					
BF1 a-1*	Apocynaceae	*Trachelospermum*	*jasminoides*	0	98.9
BF1 a-2	Apocynaceae	*Trachelospermum*	*jasminoides*	0	98.9
BF1 a-3	Pandaceae	*Galearia*	*celebica*	0	99.8
BF1 b-1	Pandaceae	*Galearia*	*celebica*	0	99.8
BF1 b-2	Combretaceae	*Terminalia*	*guyanensis*	0	100.0
BF1 b-3	Sapotaceae	*Micropholis*	*longipedicellata*	0	100.0
BF1 c-1	Moraceae	*Ficus*	*fulva*	0	100.0
BF1 c-2	Moraceae	*Ficus*	*fulva*	0	99.8
BF1 c-3	Combretaceae	*Terminalia*	*guyanensis*	0	100.0
BF2 a-1	Moraceae	*Ficus*	*subpisocarpa*	0	100.0
BF2 a-2	Moraceae	*Ficus*	*subpisocarpa*	0	100.0
BF2 a-3	Selaginellaceae	*Selaginella*	*roxburghii*	0	99.5
BF2 b-1	Fabaceae	*Butea*	*monosperma*	0	98.7
BF2 b-2	Fabaceae	*Acacia*	*tenuifolia*	0	99.5
BF2 b-3	Fabaceae	*Butea*	*monosperma*	0	98.9
BF2 c-1	Arecaceae	*Plectocomiopsis*	*geminiflora*	0	100.0
BF2 c-2	Fabaceae	*Acacia*	*tenuifolia*	0	99.5
BF2 c-3	Moraceae	*Ficus*	*subpisocarpa*	0	100.0
BF3 a-1	Burseraceae	*Canarium*	*ovatum*	0	99.8
BF3 a-2	Malvaceae	*Cola*	*acuminata*	0	100.0
BF3 a-3	Malvaceae	*Cola*	*acuminata*	0	100.0
BF3 b-1	Sapotaceae	*Micropholis*	*longipedicellata*	0	99.8
BF3 b-2	Sapotaceae	*Micropholis*	*garciniifolia*	0	100.0
BF3 b-3	Apocynaceae	*Trachelospermum*	*jasminoides*	0	98.9
BF3 c-1	Sapotaceae	*Micropholis*	*garciniifolia*	0	100.0
BF3 c-2	Sapotaceae	*Micropholis*	*garciniifolia*	0	100.0
BF3 c-3	Sapotaceae	*Micropholis*	*garciniifolia*	0	100.0
BF4 a-1	Ebenaceae	*Diospyros*	*Sp*	2.58E-133	100.0
BF4 a-2	Burseraceae	*Canarium*	*ovatum*	0	99.8
BF4 a-3	Selaginellaceae	*Selaginella*	*roxburghii*	0	99.5
BF4 b-1	Combretaceae	*Terminalia*	*guyanensis*	0	100.0
BF4 b-2	Burseraceae	*Canarium*	*ovatum*	0	99.8
BF4 b-3	Meliaceae	*Reinwardtiodendron*	*kinabaluense*	0	100.0
BF4 c-1	Erythropalaceae	*Strombosia*	*pustulata*	0	100.0
BF4 c-2	Oxalidaceae	*Dapania*	*racemosa*	0	99.3
BF4 c-3	Centroplacaceae	*Bhesa*	*paniculata*	0	100.0
**Oil palm**					
BO1 a-1	Arecaceae	*Elaeis*	*guineensis*	0	99.6

BO1 b-3	Arecaceae	*Elaeis*	*guineensis*	0	99.6
BO1 c-1	Arecaceae	*Elaeis*	*guineensis*	2.00E-92	98.0
BO2 a-1	Arecaceae	*Elaeis*	*guineensis*	0	99.0
BO2 b-1	Arecaceae	*Elaeis*	*guineensis*	0	99.0
BO2 c-2	Arecaceae	*Elaeis*	*guineensis*	0	99.0
BO3 a-1	Arecaceae	*Elaeis*	*guineensis*	3.00E-127	99.0
BO3 b-2	Arecaceae	*Elaeis*	*guineensis*	4.00E-105	100.0
BO3 c-1	Arecaceae	*Elaeis*	*guineensis*	0	99.0
BO4 b-1	Arecaceae	*Elaeis*	*guineensis*	0	99.0
**Rubber**					
BR1 a-1	Euphorbiaceae	*Hevea*	*brasiliensis*	0	100.0
BR1 b-1	Euphorbiaceae	*Hevea*	*brasiliensis*	0	100.0
BR2 a-2	Euphorbiaceae	*Hevea*	*brasiliensis*	0	100.0
BR2 c-1	Euphorbiaceae	*Hevea*	*brasiliensis*	0	99.0
BR3 a-1	Euphorbiaceae	*Hevea*	*brasiliensis*	5.42E-176	100.0
BR3 b-1	Euphorbiaceae	*Hevea*	*brasiliensis*	2.64E-161	99.7
BR3 c-1	Euphorbiaceae	*Hevea*	*brasiliensis*	1.00E-145	99.0
BR4 a-2	Euphorbiaceae	*Hevea*	*brasiliensis*	0	100.0
BR4 b-1	Euphorbiaceae	*Hevea*	*brasiliensis*	0	99.0
BR4 c-2	Euphorbiaceae	*Hevea*	*brasiliensis*	0	100.0

*small letter refer to the subplots a, b, c, number to the root number

Table S4. Molecular identification of arbuscular mycorrhizal plant host species in Harapan transformation systems. Similarity refers to species.

Plot ID	Family	Genus	Species	E-Value	Similarity
HF1 a-1	Myrtaceae	*Pimenta*	*pseudocaryophyllus*	0	99.83
HF1 a-2	Myrtaceae	*Syzygium*	sp.	0	99.82
HF1 a-3	Sapindaceae	*Nephelium*	*mutabile*	0	99.66
HF1 b-1	Dipterocarpaceae	*Shorea*	*talura*	0	99.48
HF1 b-3	Platanaceae	*Platanus*	*orientalis*	1.00E-120	98
HF1 b-4	Burseraceae	*Dacryodes*	sp.	0	99.5
HF1 c-1	Oxalidaceae	*Dapania*	*racemosa*	0	99.32
HF1 c-2	Oxalidaceae	*Dapania*	*racemosa*	0	99.26
HF1 c-3	Gnetaceae	*Gnetum*	*diminutum*	0	99.33
HF2 a-1	Sapindaceae	*Nephelium*	*mutabile*	0	99.66
HF2 a-2	Burseraceae	*Santiria*	*trimera*	0	95.11
HF2 a-3	Burseraceae	*Canarium*	*ovatum*	0	99.83
HF2 b-1	Sapotaceae	*Micropholis*	*longipedicellata*	0	100
HF2 b-2	Sapotaceae	*Micropholis*	*garciniifolia*	1.21E-129	100
HF2 b-3	Burseraceae	*Dacryodes*	sp.	0	99.49
HF2 c-1	Annonaceae	*Mitrella*	*kentia*	0	99.65
HF2 c-2	Moraceae	*Artocarpus*	*heterophyllus*	0	100
HF2 c-3	Sapotaceae	*Manilkara*	*zapota*	0	99.47
HF3 a-1	Dipterocarpaceae	*Shorea*	*acuminata*	0	99.81
HF3 a-2	Euphorbiaceae	*Agrostistachys*	*borneensis*	0	99.3
HF3 a-3	Rosaceae	*Prunus*	*brittoniana*	0	99.83
HF3 b-1	Fagaceae	*Castanopsis*	*lucida*	0	99.83
HF3 b-2	Sapotaceae	*Manilkara*	*zapota*	0	100
HF3 b-3	Flacourtiaceae	*Casearia*	*nitida*	0	99
HF3 c-1	Phyllanthaceae	*Maesobotrya*	*vermeulenii*	0	99.48
HF3 c-2	Dipterocarpaceae	*Shorea*	*tumbuggaia*	0	89.26
HF3 c-3	Burseraceae	*Trattinnickia*	*demerarae*	0	99.12
HF4 a-1	Myrtaceae	*Syzygium*	*cumini*	0	99.83
HF4 a-2	Myrtaceae	*Syzygium*	*rowlandii*	0	99.82
HF4 a-3	Myrtaceae	*Syzygium*	*rowlandii*	0	100
HF4 b-1	Fabaceae	*Spatholobus*	sp.	0	99.63
HF4 b-2	Burseraceae	*Canarium*	*oleiferum*	0	100
HF4 b-3	Burseraceae	*Canarium*	*zeylanicum*	0	99.64
HF4 c-1	Burseraceae	*Protium*	*gallicum*	0	99.29
HF4 c-2	Sapindaceae	*Nephelium*	*mutabile*	0	99.66
HF4 c-3	Dipterocarpaceae	*Shorea*	*tumbuggaia*	0	89.26
**Oil palm**					
HO1 a-3	Arecaceae	*Elaeis*	*guineensis*	2.00E-180	99
HO1 b-2	Arecaceae	*Elaeis*	*guineensis*	0	99
HO1 c-2	Arecaceae	*Elaeis*	*guineensis*	5.00E-78	100
HO2 a-4	Arecaceae	*Elaeis*	*guineensis*	1.00E-116	98

HO2 b-3	Arecaceae	*Elaeis*	*guineensis*	0	99
HO2 c-2	Arecaceae	*Elaeis*	*guineensis*	0	99
HO3 a-2	Arecaceae	*Elaeis*	*guineensis*	6.00E-170	99
HO3 b-1	Arecaceae	*Elaeis*	*guineensis*	0	99
HO3 c-3	Arecaceae	*Elaeis*	*guineensis*	0	99
HO4 a-1	Arecaceae	*Elaeis*	*guineensis*	0	99
**Rubber**					
HR1 b-3	Euphorbiaceae	*Hevea*	*brasiliensis*	0	99
HR1 c-3	Euphorbiaceae	*Hevea*	*brasiliensis*	0	99
HR2 a-3	Euphorbiaceae	*Hevea*	*brasiliensis*	0	99
HR2 c-4	Euphorbiaceae	*Hevea*	*brasiliensis*	0	99
HR3 a-1	Euphorbiaceae	*Hevea*	*brasiliensis*	0	100
HR3 b-2	Euphorbiaceae	*Hevea*	*brasiliensis*	0	100
HR3 c-1	Euphorbiaceae	*Hevea*	*brasiliensis*	0	99
HR4 a-2	Euphorbiaceae	*Hevea*	*brasiliensis*	0	100
HR4 b-2	Euphorbiaceae	*Hevea*	*brasiliensis*	0	100
HR4 c-1	Euphorbiaceae	*Hevea*	*brasiliensis*	0	100

*small letter refer to the subplots a, b, c, number to the root number.

Supplementary Table S5. Molecular identification of arbuscular mycorrhizal fungal OTUs in Bukit Duabelas National Park transformation systems. Similarity refers to species.

OTU ID	Query length	Based on MaarJam database					Deposited in NCBI database 2015		Based on NCBI database 2022		
		**Accession**	**Closest blast match**	**Query coverage**	**E-value**	**Max identity**	**Accession**	**Description**	**Closest blast match**	**Max identity**	**Accession**
**Forest**											
			**Acaulosporaceae**					**Acaulosporaceae**			
OTU_1	558	FR719957	*Acaulospora lacunosa* VTX00024	99%	0	99%	KR822761	Uncultured *Acaulospora* clone NE.CRC990.01	*Acaulospora lacunosa*	99.64 %	FR719957.2
OTU_2	562	HE610427	*Acaulospora lacunosa* 2	99%	0	99%	KR822762	Uncultured *Acaulospora* clone NE.CRC990.02	*Acaulospora lacunosa*	100%	HE610426.1
			**Ambisporaceae**					**Ambisporaceae**			
OTU_3	536	AB015712	*Ambispora leptoticha*	100%	0	100%	KR822763	Uncultured *Ambispora* clone NE.CRC990.03	*Ambispora leptoticha*	100%	AB015712.1
OTU_4	635	AJ301861	*Ambispora leptoticha* VTX00242	100%	0	100%	KR822764	Uncultured *Archaeospora* clone NE.CRC990.04	*Ambispora leptoticha*	100%	AJ301861.1
			**Archaeosporaceae**					**Archaeosporaceae**			
OTU_5	487	AF452635	*Archaeospora sp.* PODO18.1	97%	0	97%	KR822765	Uncultured *Archaeospora* clone NE.CRC990.05	*Uncultured Archaeosporaceae*	97.95 %	EU159171.1
OTU_6	618	JF414172	*Archaeospora* sp. VTX00005	95%	0	97%	KR822766	Uncultured *Archaeospora* clone NE.CRC990.06	*Glomeromycota sp.*	96.93 %	JF414172.1
			**Claroideoglomeraceae**					**Claroideoglomeraceae**			
OTU_7	577	EU340321	*Claroideoglomus* sp. NF25 VTX00193	97%	0	97%	KR822767	Uncultured *Claroideoglomus* clone NE.CRC990.07	*Claroideoglomus etunicatum*	97.05 %	MT626044.1

OTU ID	Query length	Based on MaarJam database					Deposited in NCBI database 2015		Based on NCBI database 2022		
		Accession	Closest blast match	Query coverage	E-value	Max identity	Accession	Description	Closest blast match	Max identity	Accession
OTU_8	601	HE614986	*Claroideoglomus* sp. Torrecillas12b Glo G1 VTX00193	99%	0	99%	KR822768	Uncultured *Claroideoglomus* clone NE.CRC990.08	*Claroideoglomus claroideum*	98.84 %	MN726597.1
OTU_9	634	HE615004	*Claroideoglomus sp.* Torrecillas12b Glo G3 VTX00056	97%	0	97%	KR822769	Uncultured *Claroideoglomus* clone NE.CRC990.09	uncultured *Claroideoglomus* sp.	97.00 %	HF913482.1
			**Diversisporaceae**					**Diversisporaceae**			
OTU_10	581	HE615041	*Diversispora* sp. Torrecillas12b Div2 VTX00380	98%	0	98%	KR822770	Uncultured *Diversispora* clone NE.CRC990.10	Uncultured *Diversispora* sp.	09.80 %	HE615041.1
OTU_11	588	HE615058	*Diversispora* sp. Torrecillas12b Div3 VTX00354	99%	0	99%	KR822771	Uncultured *Diversispora* clone NE.CRC990.11	Uncultured *Diversispora* sp.	100%	HE615058.1
			**Scutellosporaceae**					**Dentiscutataceae**			
OTU_12	609	FR774917	*Scutellospora heterogama* VTX00255	97%	0	97%	KR822773	Uncultured *Dentiscutata* clone NE.CRC990.12	*Dentiscutata heterogama*	97.70 %	FR774917.1
								**Racocetraceae**			
OTU_13	527	FR750215	*Scutellospora pellucida*	100%	0	100%	KR822773	Uncultured *Cetraspora* clone NE.CRC990.13	*Cetraspora pellucida*	100%	FR750215.1
			**Glomeraceae**					**Glomeraceae**			
OTU_14	592	FR821564	*Glomus* sp. Alguacil12a Glo G9 VTX00280	100%	0	100%	KR822774	Uncultured *Glomus* clone NE.CRC990.14	Uncultured *Glomus* sp.	100%	FR821564.1

OTU ID	Query length	Based on MaarJam database					Deposited in NCBI database 2015		Based on NCBI database 2022		
		Accession	Closest blast match	Query coverage	E-value	Max identity	Accession	Description	Closest blast match	Max identity	Accession
OTU_15	523	HE576928	*Glomus* sp. Alguacil12b GLO G11 VTX00149	100%	0	100%	KR822775	Uncultured *Glomus* clone NE.CRC990.15	Uncultured *Glomus* sp.	100%	HE576928.1
OTU_16	674	FR750212	*Glomus constrictum* VTX00064	97%	0	97%	KR822776	Uncultured *Funneliformis* clone NE.CRC990.16	*Funneliformis constrictum*	97.18 %	FR750212.1
OTU_17	591	FR750209	*Glomus intraradices* VTX00100	100%	0	100%	KR822777	Uncultured *Rhizophagus* clone NE.CRC990.17	*Rhizophagus intraradices*	100%	FR750209.1
OTU_18	589	DQ336493	*Glomus sp.* Kottke08-7 VTX00069	98%	0	98%	KR822778	Uncultured *Glomus* clone NE.CRC990.18	Uncultured *Glomus* sp.	98.66 %	DQ336493.1
OTU_19	651	AB546401	*Glomus* sp. VTX00084	100%	0	100%	KR822779	Uncultured *Glomus* clone NE.CRC990.19	Uncultured *Glomus* sp.	100%	AB546401.1
OTU_20	656	AB546133	*Glomus* sp. VTX00194	100%	0	100%	KR822780	Uncultured *Glomus* clone NE.CRC990.20	Uncultured *Glomus* sp.	100%	AB546133.1
OTU_21	584	AB220173	*Glomus* sp. RF1 VTX00090	100%	0	100%	KR822781	Uncultured *Glomus* clone NE.CRC990.21	*Glomus clarum*	100%	AJ852597.1
OTU_22	651	EU169414	*Glomus* sp. VTX00064	100%	0	100%	KR822782	Uncultured *Glomus* clone NE.CRC990.22	Uncultured *Glomus* sp.	100%	EU169414.1
OTU_23	591	FR821538	*Glomus* sp. Alguacil12a Glo G8 VTX00363	98%	0	98	KR822783	Uncultured *Glomus* clone	Uncultured *Glomus* sp.	98.14 %	FR821538.1
OTU_24	593	AB555664	*Glomus* sp. VTX00291	98%	0	98%	KR822784	Uncultured *Glomus* clone	Uncultured *Glomus* sp.	98.65 %	AB555664.1

OTU ID	Query length	Based on MaarJam database					Deposited in NCBI database 2015		Based on NCBI database 2022		
		**Accession**	**Closest blast match**	**Query coverage**	**E-value**	**Max identity**	**Accession**	**Description**	**Closest blast match**	**Max identity**	**Accession**
OTU_25	561	AJ430853	*Glomus* sp. Voyria symbiont type 2 VTX00126	100%	0	97%	KR822785	Uncultured *Glomus* clone NE.CRC990.25	*Glomeromycota sp*	100%	KJ952239.1
OTU_26	594	HE576915	*Paraglomus sp.* Alguacil12b Para2 VTX00350	98%	0	98%	KR822786	Uncultured *Paraglomus* clone NE.CRC990.26	Uncultured *Paraglomus* sp.	98.65 %	HE576915.1
OTU_27	592	FR693458	*Paraglomus* sp. Para2 VTX00308	98%	0	98%	KR822787	Uncultured Paraglomus clone NE.CRC990.27	Uncultured *Paraglomus* sp.	98.82 %	FR693458.1
OTU_28	594	FR848081	*Paraglomus* sp. VTX00349	98%	0	98%	KR822788	Uncultured *Paraglomus* clone NE.CRC990.28	Uncultured *Paraglomus* sp.	98.82 %	FR848081.1
**Oil palm**											
			**Acaulosporaceae**					**Acaulosporaceae**			
OTU_1	558	FR719957	*Acaulospora lacunosa* VTX00024	99%	0	99%	KR822761	Uncultured *Acaulospora* clone NE.CRC990.01	*Acaulospora lacunosa*	99.64 %	FR719957.2
OTU_2	562	HE610427	*Acaulospora lacunosa* 2	99%	0	99%	KR822762	Uncultured *Acaulospora* clone NE.CRC990.02	*Acaulospora lacunosa*	100%	HE610426.1
			**Ambisporaceae**					**Ambisporaceae**			
OTU_4	635	AJ301861	*Ambispora leptoticha* VTX00242	100%	0	100%	KR822764	Uncultured *Archaeospora* clone NE.CRC990.04	*Ambispora leptoticha*	100%	AJ301861.1
			**Glomeraceae**					**Glomeraceae**			
OTU_20	656	AB546133	*Glomus* sp. VTX00194	100%	0	100%	KR822780	Uncultured *Glomus* clone NE.CRC990.20	Uncultured *Glomus* sp.	100%	AB546133.1

OTU ID	Query length	Based on MaarJam database					Deposited in NCBI database 2015		Based on NCBI database 2022		
		Accession	Closest blast match	Query coverage	E-value	Max identity	Accession	Description	Closest blast match	Max identity	Accession
OTU_25	561	AJ430853	*Glomus* sp. Voyria symbiont type 2 VTX00126	100%	0	97%	KR822785	Uncultured *Glomus* clone NE.CRC990.25	Glomeromycota sp.	100%	KJ952239.1
**Rubber**											
			**Acaulosporaceae**					**Acaulosporaceae**			
OTU_1	558	FR719957	*Acaulospora lacunosa* VTX00024	99%	0	99%	KR822761	Uncultured *Acaulospora* clone NE.CRC990.01	*Acaulospora lacunosa*	99.64 %	FR719957.2
OTU_2	562	HE610427	*Acaulospora lacunosa* 2	99%	0	99%	KR822762	Uncultured *Acaulospora* clone NE.CRC990.02	*Acaulospora lacunosa*	100%	HE610426.1
			**Glomeraceae**					**Glomeraceae**			
OTU_15	523	HE576928	*Glomus* sp. Alguacil12b GLO G11 VTX00149	100%	0	100%	KR822775	Uncultured *Glomus* clone NE.CRC990.15	Uncultured *Glomus* sp.	100%	HE576928.1
OTU_20	656	AB546133	*Glomus* sp. VTX00194	100%	0	100%	KR822780	Uncultured *Glomus* clone NE.CRC990.20	Uncultured *Glomus* sp.	100%	AB546133.1
OTU_23	591	FR821538	*Glomus* sp. Alguacil12a Glo G8 VTX00363	98%	0	98	KR822783	Uncultured *Glomus* clone	Uncultured *Glomus* sp.	98.14 %	FR821538.1
OTU_25	561	AJ430853	*Glomus* sp. Voyria symbiont type 2 VTX00126	100%	0	97%	KR822785	Uncultured *Glomus* clone NE.CRC990.25	Glomeromycota sp.	100%	KJ952239.1

Supplementary Table 6. Molecular identification of arbuscular mycorrhizal fungal OTUs in Harapan transformation systems. Similarity refers to species.

OTU ID	Query length	Based on MaarJam database					Deposited in NCBI database 2015		Based on NCBI database 2022		
		**Accession**	**Closest blast match**	**Query coverage**	**E-value**	**Max identity**	**Accession**	**Description**	**Closest blast match**	**Max identity**	**Accession**
**Forest**											
			**Acaulosporaceae**					**Acaulosporaceae**			
OTU_1	558	FR719957	*Acaulospora lacunosa* VTX00024	99%	0	99%	KR822761	Uncultured *Acaulospora* clone NE.CRC990.01	*Acaulospora lacunosa*	99.64 %	FR719957.2
OTU_2	562	HE610427	*Acaulospora lacunosa* 2	99%	0	99%	KR822762	Uncultured *Acaulospora* clone NE.CRC990.02	*Acaulospora lacunosa*	100%	HE610426.1
			**Ambisporaceae**					**Ambisporaceae**			
OTU_4	635	AJ301861	*Ambispora leptoticha* VTX00242	100%	0	100%	KR822764	Uncultured *Archaeospora* clone NE.CRC990.04	Uncultured Archaeosporaceae sp.	0.0	100%
			**Archaeosporaceae**					**Archaeosporaceae**			
OTU_5	487	AF452635	*Archaeospora* sp. PODO18.1	97%	0	97%	KR822765	Uncultured *Archaeospora* clone NE.CRC990.05	Uncultured Archaeosporaceae	97.95 %	EU159171.1
OTU_6	618	JF414172	*Archaeospora* sp. VTX00005	95%	0	97%	KR822766	Uncultured *Archaeospora* clone NE.CRC990.06	Glomeromycota sp.	96.93 %	JF414172.1
			**Claroideoglomeraceae**					**Claroideoglomeraceae**			
OTU_7	577	EU340321	*Claroideoglomus* sp. NF25 VTX00193	97%	0	97%	KR822767	Uncultured *Claroideoglomus* clone NE.CRC990.07	*Claroideoglomus etunicatum*	97.05 %	MT626044.1
OTU_9	634	HE615004	*Claroideoglomus* sp. Torrecillas12b Glo G3 VTX00056	97%	0	97%	KR822769	Uncultured *Claroideoglomus* clone NE.CRC990.09	uncultured *Claroideoglomus* sp.	97.00 %	HF913482.1

**OTU ID**	**Query length**	**Based on MaarJam database**					**Deposited in NCBI database 2015**		**Based on NCBI database 2022**		
		**Accession**	**Closest blast match**	**Query coverage**	**E-value**	**Max identity**	**Accession**	**Description**	**Closest blast match**	**Max identity**	**Accession**
			**Diversisporaceae**					**Diversisporaceae**			
OTU_10	581	HE615041	*Diversispora* sp. Torrecillas12b Div2 VTX00380	98%	0	98%	KR822770	Uncultured *Diversispora* clone NE.CRC990.10	Uncultured *Diversispora* sp.	09.80 %	HE615041.1
OTU_11	588	HE615058	*Diversispora* sp. Torrecillas12b Div3 VTX00354	99%	0	99%	KR822771	Uncultured *Diversispora* clone NE.CRC990.11	Uncultured *Diversispora* sp.	100%	HE615058.1
								**Dentiscutataceae**			
OTU_12	609	FR774917	*Scutellospora heterogama* VTX00255	97%	0	97%	KR822773	Uncultured *Dentiscutata* clone NE.CRC990.12	*Dentiscutata heterogama*	97.70 %	FR774917.1
								**Racocetraceae**			
OTU_13	527	FR750215	*Scutellospora pellucida*	100%	0	100%	KR822773	Uncultured *Cetraspora* clone NE.CRC990.13	*Cetraspora pellucida*	100%	FR750215.1
			**Glomeraceae**					**Glomeraceae**			
OTU_15	523	HE576928	*Glomus* sp. Alguacil12b GLO G11 VTX00149	100%	0	100%	KR822775	Uncultured *Glomus* clone NE.CRC990.15	Uncultured *Glomus* sp.	100%	HE576928.1
OTU_17	591	FR750209	*Glomus intraradices* VTX00100	100%	0	100%	KR822777	Uncultured *Rhizophagus* clone NE.CRC990.17	*Rhizophagus intraradices*	100%	FR750209.1
OTU_18	589	DQ336493	*Glomus* sp. Kottke08-7 VTX00069	98%	0	98%	KR822778	Uncultured *Glomus* clone NE.CRC990.18	Uncultured Glomus	98.66 %	DQ336493.1
OTU_20	656	AB546133	*Glomus* sp. VTX00194	100%	0	100%	KR822780	Uncultured *Glomus* clone NE.CRC990.20	Uncultured *Glomus* sp.	100%	AB546133.1

OTU ID	Query length	Based on MaarJam database					Deposited in NCBI database 2015		Based on NCBI database 2022		
		**Accession**	**Closest blast match**	**Query coverage**	**E-value**	**Max identity**	**Accession**	**Description**	**Closest blast match**	**Max identity**	**Accession**
OTU_24	593	AB555664	*Glomus* sp. VTX00291	98%	0	98%	KR822784	Uncultured *Glomus*	Uncultured *Glomus* sp.	98.65 %	AB555664.1
OTU_27	592	FR693458	*Paraglomus* sp. Para2 VTX00308	98%	0	98%	KR822787	Uncultured *Paraglomus* clone NE.CRC990.27	Uncultured *Paraglomus* sp.	98.82 %	FR693458.1
**Oil palm**											
			**Acaulosporaceae**					**Acaulosporaceae**			
OTU_1	558	FR719957	*Acaulospora lacunosa* VTX00024	99%	0	99%	KR822761	Uncultured *Acaulospora* clone NE.CRC990.01	*Acaulospora lacunosa*	99.64 %	FR719957.2
OTU_2	562	HE610427	*Acaulospora lacunosa* 2	99%	0	99%	KR822762	Uncultured *Acaulospora* clone NE.CRC990.02	*Acaulospora lacunosa*	100%	HE610426.1
			**Glomeraceae**					**Glomeraceae**			
OTU_23	591	FR821538	*Glomus* sp. Alguacil12a Glo G8 VTX00363	98%	0	98	KR822783	Uncultured *Glomus* clone	Uncultured *Glomus* sp.	98.14 %	FR821538.1
OTU_25	561	AJ430853	*Glomus* sp. Voyria symbiont type 2 VTX00126	100%	0	97%	KR822785	Uncultured *Glomus* clone NE.CRC990.25	Glomeromycota sp	100%	KJ952239.1
**Rubber**											
			**Acaulosporaceae**					**Acaulosporaceae**			
OTU_1	558	FR719957	*Acaulospora lacunosa* VTX00024	99%	0	99%	KR822761	Uncultured *Acaulospora* clone NE.CRC990.01	*Acaulospora lacunosa*	99.64 %	FR719957.2
OTU_2	562	HE610427	*Acaulospora lacunosa* 2	99%	0	99%	KR822762	Uncultured *Acaulospora* clone NE.CRC990.02	*Acaulospora lacunosa*	100%	HE610426.1
								**Racocetraceae**			

OTU ID	Query length	Based on MaarJam database					Deposited in NCBI database 2015		Based on NCBI database 2022		
		**Accession**	**Closest blast match**	**Query coverage**	**E-value**	**Max identity**	**Accession**	**Description**	**Closest blast match**	**Max identity**	**Accession**
OTU_13	527	FR750215	*Scutellospora pellucida*	100%	0	100%	KR822773	Uncultured *Cetraspora* clone NE.CRC990.13	*Cetraspora pellucida*	100%	FR750215.1
			**Glomeraceae**					**Glomeraceae**			
OTU_15	523	HE576928	*Glomus* sp. Alguacil12b GLO G11 VTX00149	100%	0	100%	KR822775	Uncultured *Glomus* clone NE.CRC990.15	Uncultured *Glomus* sp.	100%	HE576928.1
OTU_23	591	FR821538	*Glomus* sp. Alguacil12a Glo G8 VTX00363	98%	0	98	KR822783	Uncultured *Glomus* clone	Uncultured *Glomus* sp.	98.14 %	FR821538.1
OTU_25	561	AJ430853	*Glomus* sp. Voyria symbiont type 2 VTX00126	100%	0	97%	KR822785	Uncultured *Glomus* clone NE.CRC990.25	Glomeromycota sp.	100%	KJ952239.1

Supplementary Method 1

Nested polymerase chain reaction (PCR) for arbuscular mycorrhizal fungi

The method was taken from Edy (2015): "Each PCR reaction (25 µL) contained: 2.5 µL 1x PCR buffer with $(NH_4)_2SO_4$ (Thermo Scientific Bio, Darmstadt, Germany), 2 µL 2 mM MgCl (Thermo Scientific Bio, Germany), 0.5 µL 0.2 mM dNTPs mix (Thermo Scientific Bio, Germany), 1.25 µL 0.5 mM of each primer, 0.125 µL 0.5 U Taq polymerase (Thermo Scientific Bio, Darmstadt, Germany), and 15.375 µL water (AppliChem GmbH, Darmstadt, Germany). The following PCR conditions were applied: initial denaturation at 94°C for 3 min, followed by 30 cycles at 94°C for 30 s, 40°C for 1 min, and 72°C for 1 min, and a final extension cycle at 72°C for 10 min. Five microliters of the resulting PCR product were used as the template for the second PCR using the specific primers, AML1 and AML2. The volume and PCR mixture was the same as described above except that the volume of water was adjusted to 12.375 µL. The PCR conditions for the second run were as follows: initial denaturation at 94°C for 3 min, followed by 30 cycles at 94°C for 1 min, 55°C for 1 min, and 72°C for 1 min, and a final extension cycle of 10 min at 72°C. Positive and negative controls using PCR-positive template and sterile water, respectively, were also included in all amplifications. All PCR reactions were run on a Mastercycler gradient (Eppendorf, Hamburg, Germany). To confirm successful PCR reactions, the PCR products (5 µL) were mixed with 1 µL 6x DNA loading buffer (3 mL glycerol, 25 mg bromophenol blue, and water to 10 mL) and separated by electrophoresis in an 1.2% agarose gel (Biozym Scientific GmbH, Oldendorf, Germany) containing 2% GelRed (Biotium, Hayward, USA) with 1x TBE electrophoresis buffer (10x TBE: 108 g Tris base [$C_4H_{11}NO_3$], 55 g boric acid in 900 mL deionized water, 40 mL of 0.5M ethylenediaminetetraacetic acid, pH 8.0, adjusted to a final volume of 1 L with deionized water) at 120 V for 60 min. PCR products, which showed a band, were purified with the innuPREP PCRpure Kit (Analytik Jena, Germany) following the manufacturer's protocol.
The purified PCR products were cloned into pGEM-T Easy vector (Promega, Madison, USA) following manufacturer's instruction and transformed into electrocompetent *E. coli* top10F' cells using the Bio-Rad *E. coli* pulser (Bio-RAD, Hercules, CA, USA). Eight positive transformants were analyzed from each sample used for cloning. Colony PCR was performed using the primer pair M13-20/M13RP (Table S2). The PCR mixture and amplification conditions were the same as described above for the second PCR run, except that the annealing temperature was set to 55°C. When PCR revealed a single clone, confirmed by the presence of single band on the gel, the product was purified by adding 35 µL 99.8% isopropanol (Roth GmbH, Karsruhe, Germany), incubating at room temperature for 60 min, and centrifuging for 30 min at 10,000× *g*. The supernatant was removed and the pellet was re-suspended in nuclease-free water (Sigma-Aldrich Corp., St. Louis, MO, USA).
In order to estimate DNA polymorphisms in the clones, restriction fragment length polymorphisms (RFLP) analysis was conducted with HinfI or BsuRI (HaeIII) following the manufacturer protocol (Life Technologies GmbH, Darmstadt, Germany). The RFLP mixtures were incubated overnight at 4°C. To observe the DNA band pattern, all RFLP products were separated on a 3% agarose gel at 90 V for 20 min followed by 120 V for 90 min as described above. Three samples of each different DNA band were sequenced (SEQLAB Sequence Laboratories Göttingen GmbH, Göttingen, Germany)."

Amplification of plant markers and sequencing

The description was taken from Edy (2015): "The PCR mixture kit (HOT FIREPol®, Tartu, Estonia) contained 1.5 µL 1x PCR buffer with B2 (Mg^{2+} free), 1.5 µL 2 mM $MgCl_2$, 1 µL 0.2 mM dNTPs mix,

0.2 µL 0.5 U DNA polymerase, 1 µL 0.5 mM of each primer, 6.8 µL water (Roth GmbH, Germany), and 1 µL 10-fold diluted DNA. The PCR conditions were as follows: initial denaturation at 95°C for 3 min, followed by 35 cycles at 94°C for 1 min, 50°C for 1 min, and 72°C for 1.5 min, and a final extension cycle at 72°C for 10 min. One set of *rbcL* primers and three sets of *matK* primers were tested for plant identification (Table S2). The three sets of *matK* primers amplified the same region at different binding sites. The primer sets were tested individually for amplification efficiency.

PCR products were cleaned using innuPREP Gel Extraction Kit (Analytik Jena, Germany). Two microliter of the resulting PCR products were used for sequencing in a total volume of 10 µL of the reaction mix with BrightDye® Terminator Cycle Sequencing Kit (Nimagen, Carlsbad, CA, USA) following manufacturer's instructions. Sequencing conditions were the same as for the plant DNA amplification described above. PCR products were purified with DyeEx Kit (Qiagen, Hilden, Germany) following manufacturer's protocol. The sequence reactions were conducted in an Applied Biosystems 3130xl Genetic Analyzer (Life Technologies GmbH, Darmstadt, Germany) in the Department of Forest Genetics and Tree Breeding, University of Göttingen)."

Edy N (2015) Community structure of arbuscular mycorrhizal fungi in temperate grassland and tropical land-use systems. PhD thesis, University of Goettingen, http://hdl.handle.net/11858/00-1735-0000-0022-608C-B, accessed 26th April, 2022.
